# Atherosclerosis is associated with plasma Aβ levels in non-hypertension patients

**DOI:** 10.1186/s12883-024-03722-z

**Published:** 2024-06-25

**Authors:** Chen Chen, Wang Anqi, Gao Ling, Wei Shan, Dang Liangjun, Shang Suhang, Huo Kang, Gao Fan, Wang Jingyi, Qu Qiumin, Wang Jin

**Affiliations:** 1 Department of Neurology, The First Affiliated Hospital of Xi’an Jiaotong University, 277 West Yanta Rd, Xi’an, 710061 China; 2 Clinical research center, The First Affiliated Hospital of Xi’an Jiaotong University, Xi’an, China; 3 Huyi Hospital of Traditional Chinese Medicine, Xi’an, China

**Keywords:** Alzheimer’s disease, Risk factors, Atherosclerosis, Plasma Aβ

## Abstract

**Background:**

Growing evidence indicated that to develop of atherosclerosis observed more often by people with Alzheimer’s disease (AD), but the underlying mechanism is not fully clarified. Considering that amyloid-β (Aβ) deposition in the brain is the key pathophysiology of AD and plasma Aβ is closely relate to Aβ deposition in the brain, in the present study, we investigated the relationships between atherosclerosis and plasma Aβ levels.

**Methods:**

This was a population based cross-sectional study. Patients with high risk of atherosclerosis from Qubao Village, Xi’an were underwent carotid ultrasound for assessment of atherosclerosis. Venous blood was collected on empty stomach in the morning and plasma Aβ_1−40_ and Aβ_1−42_ levels were measured using ELISA. Multivariate logistic regression analysis was performed to investigate the relationships between carotid atherosclerosis (CAS) and plasma Aβ levels.

**Results:**

Among 344 patients with high risk of atherosclerosis, 251(73.0%) had CAS. In the univariate analysis, the plasma Aβ levels had no significant differences between CAS group and non-CAS group (Aβ_1−40_: 53.07 ± 9.24 pg/ml vs. 51.67 ± 9.11pg/ml, *p* = 0.211; Aβ_1−42_: 40.10 ± 5.57 pg/ml vs. 40.70 pg/ml ± 6.37pg/ml, *p* = 0.285). Multivariate logistic analysis showed that plasma Aβ levels were not associated with CAS (Aβ_1−40_: OR = 1.019, 95%CI: 0.985–1.054, *p* = 0.270;Aβ_1−42_: OR = 1.028, 95%CI: 0.980–1.079, *p* = 0.256) in the total study population. After stratified by hypertension, CAS was associated with plasma Aβ_1−40_ positively (OR = 1.063, 95%CI: 1.007–1.122, *p* = 0.028) in the non-hypertension group, but not in hypertensive group. When the plasma Aβ concentrations were classified into four groups according to its quartile, the highest level of plasma Aβ_1−40_ group was associated with CAS significantly (OR = 4.465, 95%CI: 1.024–19.474, *p* = 0.046).

**Conclusion:**

Among patients with high risk of atherosclerosis, CAS was associated with higher plasma Aβ_1−40_ level in non-hypertension group, but not in hypertension group. These indicated that atherosclerosis is associated with plasma Aβ level, but the relationship may be confounded by hypertension.

## Introduction

Alzheimer’s disease (AD) is the most common cause of dementia, affecting more than 47 million people worldwide [1]. Amyloid-β (Aβ) deposition in the brain is the key pathological characteristic of AD, and the amyloid cascade hypothesis is the main pathogenesis of AD [2]. Compare to Aβ_1−42_ deposition in the brain mainly, Aβ_1−40_ deposits are mainly found in the brain and cerebral blood vessels, causing neurovascular dysfunction [3]. Aβ accumulation in the brain mainly come from the imbalance of Aβ production and clearance [4]. Aβ is generated from the cleavage of amyloid precursor protein (APP) by sequential β- and γ- secretases [5]. Aβ can be cleared from the brain into the peripheral blood through the blood-brain barrier (BBB) by LRP-1 protein, while reverse transport of peripheral Aβ across the BBB into the brain is depend on RAGE protein [6]. There is a complex dynamic equilibrium between Aβ burden in the brain and plasma Aβ [4]. Studies have shown that Aβ in the plasma is closely related to Aβ deposition in the brain [4, 5, 7].

Atherosclerosis is an important risk factor for ischemic stroke. Growing evidence have indicated that atherosclerosis is also associated with AD [8, 9]. Atherosclerosis could disrupt the structure and function of cerebrovascular, and induce hypoperfusion and hypoxia, which may promote the production of Aβ in the brain [10]. Meanwhile Aβ may contribute to ischemic brain even to atherosclerotic lesions through vascular oxidative stress and endothelial dysfunction [11]. However, the relationships between atherosclerosis and AD is not determined fully.

Carotid atherosclerosis (CAS) is a hallmark for atherosclerosis [12] and is closely associated with ischemic stroke [13, 14]. A large body of evidence suggested that CAS is associated with cognitive decline [15–18] as the patients with AD. In the present study, we investigated the relationships between CAS and plasma Aβ in patients with high-risk atherosclerosis.

## Methods

### Participants

This was a population based cross-sectional study. All participants came from Qubao village, Huyi district of Xi’an. The village was selected using clustering sampling method. The inclusion criteria was follow: (1) permanent resident who living in the village for more than 3 years; (2) aged 40 years or older (3)People who have at least 3 risk factors in the follow 8 items: smoking, less exercise, hypertension, diabetes, atrial fibrillation, hyperlipidemia, stroke family history and obesity. (4) Agree to participate in the study and signed written informed consent. Exclusion criteria: (1) Did not complete carotid ultrasound; (2) Missing data of plasma Aβ or outliers of plasma Aβ.;(3) With liver and kidney dysfunction. This study protocol was approved by the Ethics Committee of the First Affiliated Hospital of Xi’an Jiao tong University (No. XJTU1AF2014LSK-111).

### Data collection

All participants received a face-to-face interview to complete a standardized questionnaires for general information (age, sex, education levels and jobs) and lifestyle habits (alcohol abuse, smoking history, physical activity level, sleep), the medical history (hypertension, diabetes, hyperlipidaemia, cardiovascular disease, transient ischaemic attack, stroke), and underwent a systemic and neurologic examination to measure height, weight, blood pressure and the pulse rate. Body mass index (BMI) was defined as a person’s weight in kilograms divided by the square of his or her height in meters (kg/m^2^). The questionnaire used in study was developed for a sequential design to determine the potential vascular factors for AD in the general population [19].

After fasting overnight, 3 ml cubital venous blood were collected from all subjects between 8 and 10 am, and placed in an EDTA anticoagulant tube, centrifuged at 3000 g for 10 min at room temperature (20℃), and had the supernatant plasma extracted and aliquoted. Aliquots of plasma were stored at -80℃ pending biochemical analyses. Laboratory test parameters were measured in the clinical laboratory of the First Affiliated Hospital of Xi’an Jiaotong University.

### Atherosclerosis assessment

High-resolution B-mode ultrasonography with a linear-array, 5 to 10 MHz transducer was used to assess CAS. The probe scanned the proximal, middle and distal common carotid arteries, the carotid sinus, internal carotid artery, external carotid artery, and measured carotid artery, internal carotid artery, external carotid artery diameter, intima-media thickness (IMT). IMT is the distance between the lumen membrane interface and the media-adventitia interface. The diagnosis of CAS is: (1) IMT ≥ 1.0 mm. (2) Had a plaque in carotid artery, including common carotid arteries, carotid sinus, internal carotid artery, external carotid artery.

### Quantification of plasma Aβ

The levels of plasma Aβ_1−40_ and Aβ_1−42_ were measured as previously described [20]. Briefly, double-antibody sandwich enzyme-linked immunoassay (ELISA) was used to determine plasma Aβ concentrations. The kit was purchased from Shanghai Yuanye Biotechnology Co., Ltd. All samples were measured in duplicate using an RT-6000 analyzer from Rayto Co. based in Shenzhen, China. The measurements were taken at a wavelength of 450 nm, and the procedures followed were strictly in accordance with the instructions provided. The concentration was calculated based on the standard curve, and the average was taken as the sample concentration.

### Definition of covariates

The diseases involved in this study are defined as previously described [21].Hypertension was defined as follows: a mean systolic blood pressure measurement ≥ 140mmHg or a diastolic blood pressure ≥ 90mmHg, self-reported medical diagnosis, or the usage of antihypertensive drugs. The definition of diabetes mellitus included the following criteria: fasting blood glucose (FBG) ≥ 7.0mmol/L, the usage of diabetic medication or insulin. The definition of hyperlipidaemia included the following criteria: serum cholesterol concentration (TC) > 5.18 mol/L, serum triglyceride concentration (TG) > 1.70 mmol/L, low density lipoprotein cholesterol (LDL-c) > 3.37 mmol/L, high density lipoprotein cholesterol (HDL-c) < 1.04 mmol/L, self-reported medical diagnosis, or the usage of lipid-lowering drugs.

### Statistical analyses

SPSS version 24.0 (SPSS Inc., IBM, Chicago) was used to perform statistical analyses. Continuous variables with approximately normal distribution were expressed as means ± standard deviations (SDs). The data of skewed distributions were described as the median (25% percentile, 75% percentile). Percentages were used for categorical variables. *p* < 0.05 was identified as a significant difference.

For univariate analyses, one-way analysis of variance, unpaired Student’s t-test, Mann-Whitney U test, and *χ*^*2*^test (the chi-squared test) were chosen to compare the different types of variables. About one-way analysis of variance test, homogeneity of variances of the variables was confirmed with Levene’s test, and the comparison between any two groups was performed with least significant difference test. Then, for multivariate analysis, multiple linear regression (MLR) analysis was used to analyze the relationship between CAS and plasma Aβ_1−40_, Aβ_1−42_, Aβ_1−42_/Aβ_1−40_ levels. In the MLR models, Aβ_1−40_, Aβ_1−42_ concentrations were the dependent variables, respectively. Other covariates were independent variables, adjusted factors including age, sex, BMI, medical history of stroke, diabetes, coronary heart disease, dyslipidemia, lack of exercise, smoking, and drinking, FBG, TG, TC, LDL-c, HDL-c.

## Results

### Demographics and clinical characteristics

**Fig. 1:**
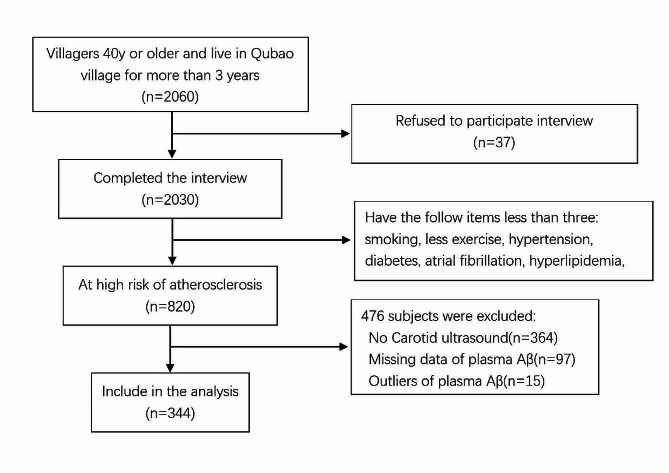
The enrollment flow chart of this study

There were 2060 in the population, among which 820 patients met the criteria for high risk of atherosclerosis, 364 did not complete carotid ultrasonography, 97 did not have plasma Aβ, 15 had outliers of Aβ levels, finally 344 patients included in the analysis. (Fig. 1)

Among 344 participants, 251(73.0%) had CAS. Compare to non-CAS group, CAS group was older (61.54 ± 9.01 years vs. 51.05 ± 7.03 years, *p*<0.0001), and had more hypertension (60.6% vs. 47.3%, *p* = 0.028), diabetes mellitus (32.3% vs. 16.1%, *p* = 0.003), stroke (35.5% vs. 15.1%, *p*<0.0001) and less dyslipidemia (70.1% vs. 81.7%, *p* = 0.031). CAS group had lower BMI (26.84 ± 3.03 vs. 27.81 ± 3.08, *p* = 0.009) and higher HDL-c (1.37 ± 0.33 vs. 1.28 ± 0.28, *p* = 0.032), while others had no significant difference between the two groups. (Table 1)

**Table 1:** Demographic data and clinical characteristics of the study population

Variable	Total (*n* = 344)	Non-CAS group (*n* = 93)	CAS group (*n* = 251)	t/u/chi square	*p*
Age (y, mean ± SD)	58.7 ± 9.70	51.05 ± 7.03	61.54 ± 9.01	-10.134	**<0.0001**
Female (n, %)	160(46.5%)	41(44.1%)	119(47.4%)	0.301	0.583
BMI (kg/m², mean ± SD)	27.10 ± 3.07	27.81 ± 3.08	26.84 ± 3.03	2.619	**0.009**
Smoking (n, %)	148(43.0%)	45(48.4%)	103(41.0%)	0.356	0.551
Drinking (n, %)	58(16.9%)	18(19.4%)	40(15.9%)	0.566	0.452
Lack of Exercise (n, %)	99(28.8%)	22(23.7%)	77(30.7%)	1.632	0.201
Hypertension (n, %)	196(57.0%)	44(47.3%)	152(60.6%)	4.857	**0.028**
Diabetes mellitus (n, %)	96(27.9%)	15(16.1%)	81(32.3%)	8.788	**0.003**
Coronary heart disease (n, %)	43(12.5%)	10(10.8%)	33(13.1%)	0.356	0.551
Stroke (n, %)	103(29.9%)	14(15.1%)	89(35.5%)	13.468	**<0.0001**
Dyslipidemia (n, %)	252(73.3%)	76(81.7%)	176(70.1%)	4.661	**0.031**
FBG (mmol/L, Median)	5.95(5.00, 6.10	5.75(5.15, 6.46)	6.40(5.17, 6.65)	13732.5	0.068
TC (mmol/L, mean ± SD)	5.37 ± 1.16	5.36 ± 1.03	5.38 ± 1.21	-0.137	0.891
TG (mmol/L, Median)	2.02(1.31, 2.38)	2.29(1.46, 2.84)	1.84(1.34, 2.52)	9768	0.065
HDL-c(mmol/L, mean ± SD)	1.34 ± 0.32	1.28 ± 0.28	1.37 ± 0.33	-2.149	**0.032**
LDL-c(mmol/L, mean ± SD)	3.62 ± 1.02	3.61 ± 0.93	3.62 ± 1.05	-0.032	0.975
Aβ_1−40_ (pg/ml, mean ± SD)	52.69 ± 9.22	51.67 ± 9.11	53.07 ± 9.24	-1.253	0.211
Aβ_1−42_ (pg/ml, mean ± SD)	40.70 ± 6.37	40.10 ± 5.57	40.09 ± 5.57	-1.071	0.285
Aβ_1−40_/Aβ_1−42_ (mean ± SD)	1.33 ± 0.33	1.32 ± 0.32	1.33 ± 0.34	-0.465	0.642

Independent sample t-test and mean ± SD were used to compare the difference of the approximately normally distributed continuous variables between the CAS group and the non-CAS group. Mann-Whitney U test and median (quartile) were used for the skew distributional data and Chi square and percentage were used for categorical variables. Data are mean (SD), median (interquartile range), or number (percentage). The skew distributional data include FBG and TG. BMI, body mass index; FBG, fast blood glucose; TC, total cholesterol; TG, triglyceride; HDL-c, high-density lipoprotein cholesterol; LDL-c, low-density lipoprotein cholesterol; CAS, carotid atherosclerosis

### Comparison of plasma Aβ levels between CAS group and non-CAS group

As shown in Table 1, the level of plasma Aβ_1−40_ and Aβ_1−42_ had no differences between CAS group and non-CAS group (Aβ_1−40_: 53.07 ± 9.24 pg/ml vs. 51.67 ± 9.11 pg/ml, *p* = 0.211; Aβ_1−42_: 40.09 ± 5.57 pg/ml vs. 40.10 ± 5.57 pg/ml, *p* = 0.285). Also, the Aβ_1−40_/Aβ_1−42_ ratio had no differences between CAS group and non-CAS group (1.33 ± 0.34 vs. 1.32 ± 0.32, *p* = 0.642).

### The relationships between CAS and plasma Aβ in total study population

To investigate the relationships between CAS and plasma Aβ levels, a multiple linear regression analysis was used, as plasma Aβ levels as the dependent variable, and CAS as covariates. As shown in Table 2, plasma Aβ_1–40_ was not related with CAS in unadjusted MLR model, and in MLR model adjusted for age, sex, BMI, medical history of stroke, diabetes, coronary heart disease, dyslipidemia, lack of exercise, smoking, and drinking, FBG, TG, TC, LDL-c, HDL-c. Also. plasma Aβ1–42 was not associated with CAS in unadjusted MLR model and adjusted MLR model.

**Table 2:** Multiple linear regression analysis of Aβ and CAS in total study population

	Aβ_1−40_			Aβ_1−42_		
	OR	95%CI	*p*	OR	95%CI	*p*
Model 1	1.017	0.991–1.044	0.198	1.022	0.984–1.061	0.267
Model 2	1.019	0.988–1.052	0.235	1.016	0.971–1.063	0.493
Model 3	1.016	0.983–1.051	0.344	1.026	0.979–1.076	0.276
Model 4	1.019	0.985–1.054	0.270	1.028	0.980–1.079	0.256

Model 1 is unadjusted. Model 2 is adjusted for age and gender. Model 3 is adjusted for model 2 and BMI, medical history of hypertension, stroke, diabetes, coronary heart disease, dyslipidemia, lack of exercise, smoking, and drinking. Model 4 is adjusted for model 3 and fasting blood glucose, total cholesterol, triglyceride, low-density lipoprotein, high-density lipoprotein

### Comparison between hypertension group and no-hypertension

As hypertension is an important risk factor for atherosclerosis, and also is associated with plasma Aβ levels, we stratified the participants into hypertension group (*n* = 196) and non- hypertension (*n* = 148). As shown in Table 3, compared to non-hypertension group, hypertension group are older, had more females, diabetes mellitus, cardiovascular disease and stroke, CAS, drinking, BMI. The levels of plasma Aβ_1−40_ and Aβ_1−42_ had no significant differences between hypertension group and non-hypertension group.

**Table 3:** General characteristics of the total study population

Variable	Non-Hypertension group (*n* = 148)	Hypertension group (*n* = 196)	*p*
Age (y, mean ± SD)	56.85 ± 10.37	60.10 ± 8.94	**0.002**
Female (n, %)	44 (29.7%)	116 (59.2%)	**<0.0001**
Diabetes mellitus (n, %)	31(20.9%)	65(33.2%)	**0.012**
Cardiovascular disease (n, %)	10(23.3%)	33(76.7%)	**0.005**
Stroke (n, %)	35(34.0%)	68(66.0%)	**0.027**
Smoking (n, %)	85(57.4%)	63(42.6%)	**<0.0001**
FBG (mmol/L, Median)	5.47(5.05,6.40)	5.61(5.18,6.53)	0.904
Lack of Exercise (n, %)	44(44.4%)	55(55.6%)	0.735
Drinking (n, %)	35(60.3%)	23(39.7%)	**0.003**
dyslipidemia (n, %)	101(40.1%)	151(59.9%)	0.068
Carotid atherosclerosis (n, %)	99(39.4%)	152(60.6%)	**0.028**
BMI (kg/m^2^, mean ± SD)	26.66 ± 2.90	27.43 ± 3.16	**0.021**
TC (mmol/L, mean ± SD)	5.35 ± 1.12	5.39 ± 1.19	0.760
TG (mmol/L, Median)	1.87(1.30,2.58)	1.82(1.37,2.45)	0.924
HDL-c (mmol/L, mean ± SD)	1.33 ± 0.34	1.36 ± 0.31	0.419
LDL-c (mmol/L, mean ± SD)	1.36 ± 0.31	3.63 ± 1.04	0.842
Aβ_1−40_ (pg/ml, mean ± SD )	52.44 ± 9.32	52.89 ± 9.15	0.654
Aβ_1−42_ (pg/ml, mean ± SD)	40.81 ± 6.52	40.61 ± 6.26	0.774
Aβ_1−40_ / Aβ_1−42_ (mean ± SD)	1.32 ± 0.34	1.33 ± 0.33	0.692

The skew distributional data include FBG and TG. BMI, body mass index; FBG, fast blood glucose; TC, total cholesterol; TG, triglyceride; HDL-c, high-density lipoprotein cholesterol; LDL-c, low-density lipoprotein cholesterol; CAS, carotid atherosclerosis

After stratified by hypertension, in the non-hypertension group, plasma Aβ_1−42_ was higher in CAS group than that in non-CAS group (41.50 ± 7.01 pg/ml vs. 39.42 ± 5.18 pg/ml, *p* = 0.012), but plasma Aβ_1−40_ had no significant differences between CAS group and non-CAS group (53.53 ± 9.49 pg/ml vs. 50.67 ± 8.79 pg/ml, *p* = 0.391). In the hypertension group, both plasma Aβ_1−40_ and Aβ_1−42_ had no difference between CAS group and non-CAS group. (Table 4)

The Aβ_1–40_ (53.11 ± 8.91 pg/ml vs. 50.25 ± 10.38 pg/ml, *p* = 0.088) and Aβ_1–42_(40.78 ± 6.60 pg/ml vs. 40.93 ± 6.34 pg/ml, *p* = 0.351) levels had no significant difference between non-stroke group and stroke groups in the non-hypertensive group. Also, in the non-hypertensive group, the Aβ_1–40_ (52.07 ± 9.40 pg/ml vs. 54.42 ± 8.52 pg/ml, *p* = 0.112) and Aβ_1–42_(40.92 ± 6.06 pg/ml vs. 40.04 ± 6.63 pg/ml, *p* = 0.351) levels had no significant difference between non-stroke group and stroke group.

**Table 4:** Plasma levels of Aβ1–40 and Aβ1–42 in total study population

	Non-Hypertension group			Hypertension group				
	Aβ_1−40_ (pg/ml)	Aβ_1−42_ (pg/ml)	Aβ_1−40_ / Aβ_1−42_		Aβ_1−40_ (pg/ml)	Aβ_1−42_ (pg/ml)	Aβ_1−40_ / Aβ_1−42_	
Non-CAS group	50.67 ± 8.79	39.42 ± 5.18	1.31 ± 0.31		52.79 ± 9.42	40.85 ± 5.95	1.32 ± 0.32	
CAS group	53.53 ± 9.49	41.50 ± 7.01	1.33 ± 0.35		52.92 ± 9.10	40.55 ± 6.37	1.34 ± 0.33	
*p*	0.391	**0.012**	0.821		0.638	0.355	0.714	

Independent sample t test was used to compare plasma Aβ levels between CAS group and non-CAS group

### Multivariate logistic regression analysis of plasma Aβ and CAS after stratified by hypertension

To exclude the effects of covariates on the relationships between CAS and plasma Aβ levels, a multivariate logistic analysis stratified by hypertension was used. CAS is as the dependent variable (yes or no), and plasma Aβ levels (Aβ_1−40_, Aβ_1−42_) and covariates are as independent variables to establish a logistic regression model. Covariates were chosen according to previously described in univariate analyses as well as covariates reported to be related to cognition in previous studies.

In the non-hypertension group, plasma Aβ_1–40_ did not associate with CAS in unadjusted MLR model, but significantly associate with CAS in MLR Models adjusted for all covariates which include age, sex, BMI, medical history of stroke, diabetes, coronary heart disease, dyslipidemia, lack of exercise, smoking, and drinking, FBG, TG, TC, LDL-c, HDL-c.(OR = 1.063, 95%CI: 1.007–1.122, *p* = 0.028). (Table 5）However, plasma Aβ_1–42_ did not associate with CAS in MLR models whenever they are unadjusted or adjusted for covariates.

In the hypertension group, both plasma Aβ_1–40_ and Aβ_1–42_ were not associated with CAS in unadjusted Models and adjusted Models for all covariates. (Table 6)

**Table 5:** Logistic regression analysis of plasma Aβ and CAS in non-hypertension group

		Aβ_1−40_		Aβ_1−42_		
	OR	95%CI	*p*	OR	95%CI	*p*
Model 1	1.035	0.995–1.075	0.084	1.056	0.999–1.117	0.055
Model 2	1.063	1.011–1.117	**0.017**	1.065	0.990–1.145	0.090
Model 3	1.063	1.008–1.121	**0.024**	1.072	0.992–1.158	0.080
Model 4	1.063	1.007–1.122	**0.028**	1.073	0.991–1.163	0.082

Model 1 is unadjusted. Model 2 is adjusted for age and gender. Model 3 is adjusted for model 2 and BMI, medical history of stroke, diabetes, coronary heart disease, dyslipidemia, lack of exercise, smoking, and drinking. Model 4 is adjusted for model 3 and fasting blood glucose, total cholesterol, triglyceride, low-density lipoprotein, high-density lipoprotein

**Table 6:** Logistic regression analysis of plasma Aβ and CAS in hypertension group

	Aβ_1−40_			Aβ_1−42_		
	OR	95%CI	*p*	OR	95%CI	*p*
Model 1	1.001	0.965–1.039	0.938	0.992	0.941–1.047	0.780
Model 2	0.987	0.945–1.031	0.554	0.985	0.928–1.046	0.629
Model 3	0.959	0.911–1.011	0.118	0.987	0.923–1.056	0.707
Model 4	0.966	0.918–1.016	0.180	0.989	0.923–1.060	0.758

Model 1 is unadjusted. Model 2 is adjusted for age and gender. Model 3 is adjusted for model 2 and BMI, medical history of stroke, diabetes, coronary heart disease, dyslipidemia, lack of exercise, smoking, and drinking. Model 4 is adjusted for model 3 and fasting blood glucose, total cholesterol, triglyceride, low-density lipoprotein, high-density lipoprotein

According to plasma Aβ concentrations, non-hypertension group were classified into four quartiles. Multivariate logistic regression analysis showed that the Quartile 1 (the lowest level of the plasma Aβ_1−40_) group as the reference, the Quartile 4 (the highest level of plasma Aβ_1−40_) group was obviously associated with CAS (OR = 4.465, 95%CI: 1.024–19.474, *p* = 0.046), but plasma Aβ_1−42_ did not. (Table 7)

**Table 7:** Multivariate Logistic Regression Analysis of CAS and Different Levels of Plasma Aβ

variables	B	S.E	Wald	df	*p*	Exp(B)	95%CI
Aβ_1−40_							
Quartile 1	reference						
Quartile 2	0.274	0.802	0.116	1	0.733	1.315	0.273–6.332
Quartile 3	1.022	0.617	2.744	1	0.098	2.780	0.829–9.318
Quartile 4	1.496	0.751	3.965	1	**0.046**	4.465	1.024–19.474
Aβ_1−42_							
Quartile 1	reference						
Quartile 2	− 0.063	0.637	0.010	1	0.921	0.939	0.270–3.271
Quartile 3	− 0.137	0.676	0.041	1	0.840	0.872	0.232–3.282
Quartile 4	1.351	0.725	3.473	1	0.062	3.860	0.933–15.979

Model is adjusted for age, gender and medical history of stroke, coronary heart disease, lack of exercise, smoking, and drinking, BMI, fasting blood glucose, total cholesterol, triglyceride, low-density lipoprotein, high-density lipoprotein

## Discussion

In this population based cross-sectional study, we found that plasma Aβ levels had no significant difference between CAS group and non-CAS group in the population with high risk of atherosclerosis. However, in the stratified multiple analyses, we found that CAS was associated with higher plasma Aβ_1−40_ level in non-hypertension group, but not in hypertension group.

The relationship between atherosclerosis and AD has not been determined. Due to plasma Aβ closely relate to Aβ deposition in the brain, several studies have explored the relationships between atherosclerosis and plasma Aβ. One study found that Aβ_1−40_ deposits in CAS plaques and in the aorta [22]. Subclinical atherosclerosis patients and CAD-stabilized patients have higher levels of circulating Aβ_1−40_, which predict cardiovascular mortality and major adverse cardiac events [23]. Cohort study showed that plasma Aβ_1−40_ level was significantly associated with arterial stiffness progression, subclinical atherosclerotic events, and coronary heart disease events [24]. In the present study, we used a cluster sampling method to select study population and all people who living in the village were included in the analysis. To make sure the enough individuals with atherosclerosis for analyses, we enrolled the patients with high risk of atherosclerosis. As CAS is easily and exactly detected using B-mode ultrasonography, and is closely relate to ischemic stroke, so we detected CAS to indicating atherosclerosis. The results showed that the prevalence of CAS was 73.0% in our study group.

It is known that hypertension is the most important risk factor for atherosclerosis. In the previous study, we found that elevated blood pressure was associated with increased plasma Aβ_1-40_ level in middle-aged and elderly [25]. So, we did stratified analysis by hypertension, and found that CAS group had higher plasma Aβ_1-40_ level in non-hypertension group. The results were demonstrated in the multiple analyses after adjusted for covariates. These suggested that the association between CAS and plasma Aβ_1-40_ level was confounded by hypertension.

The reason of CAS relate to plasma Aβ level have not been clarified fully. Many animal and cell studies have shown that Aβ_1−40_ is involved in the formation of atherosclerosis through various channels. Knockout of Herpud1 reduced Aβ_1−40_ expression other than lipid metabolism and alleviated atherosclerosis via JNK/AP1 signaling inhibition [26]. Neuronal overexpression of Aβ in atherosclerotic mice predisposes to macrophage activation and endothelial dysfunction, which promotes atherosclerosis [27]. Li found a positive correlation between brain Aβ loading and aortic fat streak formation, and suggested that local Aβ overexpression in the brain maintains the inflammatory response and progression of atherosclerosis in mouse models by developing endothelial dysfunction and atherosclerosis [28]. Puglielli suggests that Aβ-catalyzed the formation of 4-chosterane-3-ketones may be responsible for the increase in atherosclerosis in Tg2576 transgenic mice [29]. In contrast, experimental study [30] have shown that spatial memory was significantly affected 3 weeks after calcification using a mouse model based on carotid artery calcification. Carotid artery stiffness did not affect the production of Aβ or tau phosphorylation in mice, but resulted in a modest increase in the proportion of Aβ_1−40_/Aβ_1−42_ in the frontal cortex.

In the Rotterdam study [31]^,^ they found that patients with severe atherosclerosis had higher risk for AD compared with patients without atherosclerosis. A systematic review showed that [32]^,^ peripheral arterial disease was associated with dementia and cognitive impairment. The carotid artery stenosis had a higher risk for dementia and for cognitive impairment. These all suggest that atherosclerosis may play a role in the progression of dementia, the underlying mechanism may associate with the effects of plasma Aβ on carotid atherosclerosis.

Atherosclerotic vascular disease and AD have a common pathophysiological involved in inflammation, macrophage infiltration, and vascular obstruction despite their different end-stage manifestations [33]. Study has shown that [34] atherosclerotic lesions in the elderly contain a heterogeneous mixture of Aβ peptides. The source of these Aβ peptides may be vascular wall cells expressing APP/PN2/Aβ, as well as platelets involved in atherosclerotic inflammation and perturbation coagulation cascades. De Meyer and colleagues clearly showed the presence of APP and Aβ in platelets engulfed by macrophages in the neo vascular-forming region of advanced atherosclerotic lesions [35]. The presence of Aβ peptides stimulated by iNOS and COX-2 production, increase activation of macrophages and sustain synthesis of pro-inflammatory-related factors. Aβ accumulation induces microvascular inflammation mediated by cytokines and chemokines that act on neurons, glial, endothelial cells, and muscle cells [36, 37]. These microvascular inflammations eventually lead to the destruction of the vascular walls. The presence of Aβ peptides in atherosclerotic plaques may synergistically increase the chronic inflammatory process that maintains degeneration and destruction of the arterial wall.

CAS associated with plasma Aβ_1−40_ in the non-hypertensive group, but not hypertension group, suggesting that the relationship between Aβ_1−40_ and CAS was confused by blood pressure. Hypertension is a common risk factor for stroke and AD [38, 39]. Hypertension has been shown to worsen Aβ-induced neurovascular dysfunction and promote β secretase activity, these lead to an increase in Aβ production, which may contribute to pathogenic interactions between hypertension, stroke and AD [40]. Hypertension is associated with cognitive impairment and pathological features of AD, including nerve fiber tangles and Aβ deposition [41]. Our previous study found that the increase in PP is associated with an increase in plasma Aβ_1−40_ and a decrease in the soluble advanced glycation end product-specific receptor (sRAGE), as well as an increase in blood pressure associated with an increase in plasma Aβ_1−40_ levels in middle-aged and elderly ApoEε4 non-carriers. Its underlying mechanism may be related to peripheral Aβ clearance. In addition, under normal blood pressure, the walls of the blood vessels are not damaged, and the transport of Aβ is not restricted. However, in hypertension, blood vessels may be damaged, so the transport of Aβ peptides worsens. Thus, in the normal blood pressure group was able to more realistically reflect the relationship between plasma Aβ and CAS.

There were several limitations should be noted. First, this is a population based cross-sectional study, we did not follow-up the changes of plasma Aβ, and did determine the cause relationship between CAS and plasma Aβ. Second, due to smaller sample size, we did not analyze the relationships between CAS degree and plasma Aβ levels. We are not sure whether sever CAS has more changes of plasma Aβ level. Third, the deposition of Aβ in the brain has not been simultaneously detected.

## Conclusions

In summary, through this population based cross-sectional study, we found that in patients with higher risk of atherosclerosis., CAS was associated with higher plasma Aβ_1−40_ level in non-hypertension group but not in hypertension group. These indicated that atherosclerosis was associated with plasma Aβ level, but the relationship may be confounded by hypertension.

## Data Availability

No datasets were generated or analysed during the current study.
